# Time to clinical improvement: an appropriate surrogate endpoint for pulmonary arterial hypertension medication trials

**DOI:** 10.3389/fcvm.2023.1142721

**Published:** 2023-06-12

**Authors:** An Wang, Mengqi Chen, Qi Zhuang, Lihua Guan, Weiping Xie, Lan Wang, Wei Huang, Zhaozhong Cheng, Shiyong Yu, Hongmei Zhou, Jieyan Shen

**Affiliations:** ^1^ Department of Cardiology, Renji Hospital, School of Medicine, Shanghai Jiao Tong University, Shanghai, China; ^2^ Department of Cardiology, Shanghai Institute of Cardiovascular Disease, Zhongshan Hospital, Fudan University, Shanghai, China; ^3^ Department of Respiratory and Critical Care Medicine, The First Affiliated Hospital of Nanjing Medical University, Nanjing, China; ^4^ Department of Cardio-Pulmonary Circulation, Shanghai Pulmonary Hospital, School of Medicine, Tongji University, Shanghai, China; ^5^ Department of Cardiology, The First Affiliated Hospital of Chongqing Medical University, Chongqing, China; ^6^ Respiratory Department, The Affiliated Hospital of Qingdao University, Qingdao, China; ^7^ Department of Cardiology, The Second Affiliated Hospital, Third Military Medical University (Army Medical University), Chongqing, China; ^8^ Congenital Heart Disease Center, Wuhan Asia Heart Hospital, Wuhan University of Science and Technology, Wuhan, China

**Keywords:** pulmonary arterial hypertension, time to clinical improvement, efficacy, risk stratification, 6-min walk distance

## Abstract

**Background:**

Many retrospective studies suggest that risk improvement may be a suitable efficacy surrogate endpoint for pulmonary arterial hypertension (PAH) medication trials. This prospective multicenter study assessed the efficacy of domestic ambrisentan in Chinese PAH patients and observed risk improvement and time to clinical improvement (TTCI) under ambrisentan treatment.

**Methods:**

Eligible patients with PAH were enrolled for a 24-week treatment with ambrisentan. The primary efficacy endpoint was 6-min walk distance (Δ6MWD). The exploratory endpoints were risk improvement and TTCI, defined as the time from initiation of treatment to the first occurrence of risk improvement.

**Results:**

A total of 83 subjects were enrolled. After ambrisentan treatment, Δ6MWD was significantly increased at week 12 (42.2 m, *P *< 0.0001) and week 24 (53.4 m, *P *< 0.0001). Within 24 weeks, risk improvement was observed in 53 (64.6%) subjects (*P *< 0.0001), which is higher than WHO-FC (30.5%) and TAPSE/PASP (32.9%). Kaplan–Meier analysis of TTCI showed a median improvement time of 131 days and a cumulative improvement rate of 75.1%. Also, TTCI is consistent across different baseline risk status populations (log-rank *P* = 0.51). The naive group had more risk improvement (*P* = 0.043) and shorter TTCI (log-rank *P* = 0.008) than the add-on group, while Δ6MWD did not show significant differences between the two groups.

**Conclusions:**

Domestic ambrisentan significantly improved the exercise capacity and risk status of Chinese PAH patients. TTCI has a relatively high positive event rate within 24-week treatment duration. Compared to Δ6MWD, TTCI is not affected by baseline risk status. Additionally, TTCI could identify better improvements in patients, which Δ6MWD does not detect. TTCI is an appropriate composite surrogate endpoint for PAH medication trials.

**Clinical Trial Registration:**

NCT No. [ClinicalTrials.gov], identifier [NCT05437224].

## Introduction

Pulmonary arterial hypertension (PAH) is a group of diseases characterized by persistent, progressive small pulmonary artery remodeling and elevated pulmonary vascular resistance. Targeted medical therapy is the primary treatment for PAH, with the goal of achieving low-risk stratification status (1). Current surrogate endpoints to assess the efficacy of PAH medications include a change in 6-min walk distance (Δ6MWD) in the short term (2, 3) and time to clinical worsening (TTCW) in the long term (4, 5). However, Δ6MWD is poorly correlated to long-term outcomes, leading to the controversy among researchers (6–10). Also, the limited occurrence of positive events restricts TTCW from being used in small-sample or short-term trials (11). Additionally, they are not directly related to the treatment goal of achieving low-risk status. A more appropriate surrogate endpoint is still needed.

The 6th World Symposium on Pulmonary Hypertension (6thWSPH) advocates the use of time to clinical improvement (TTCI) and risk stratification tools as surrogate endpoints for phase 2–3 studies (12, 13). The latest ESC/ERS PAH guidelines adopt a new four-strata risk model (COMPERA2.0) for managing PAH patients (1, 14, 15). In addition, Hoeper et al. validated that subjects who showed improvement in risk stratification had better survival rates than those who did not show improvement (16). These studies indicated that improving risk stratification or achieving low-risk status may be suitable surrogate endpoints for efficacy trials. However, no prospective studies have been conducted to observe these ideas, nor have studies considering the timing of improvement occurrences. In this prospective study, we defined TTCI by two risk-related methods and observed which is more appropriate in short-term trials.

Ambrisentan, a selective endothelin receptor A antagonist, is a first-line agent for treating patients with PAH (17, 18). A domestic ambrisentan has been marketed in the Chinese mainland after bioequivalence testing but has not undergone a formal clinical efficacy study. The purpose of this prospective multicenter study is to investigate the efficacy of domestic ambrisentan in treating Chinese adults with PAH and to observe risk improvement and two kinds of TTCI characteristics under ambrisentan treatment.

## Methods

### Study design

This is a prospective, open-label, multicenter, single-arm cohort study conducted from September 2018 to February 2022 at eight centers in China. After a 4-week screening period, all eligible patients will receive 5 mg of ambisentan once daily for a 12-week initial treatment period. This is followed by a 12-week extension treatment period during which the 5 mg dose will be maintained or the dose will be increased up to 10 mg once daily. The domestic ambrisentan used in this study is provided by China Jiangsu Hansoh Pharmaceutical Group Co.

### Study population

Chinese subjects aged 18–75 years, diagnosed with group 1 PAH of WHO-updated clinical classification (19) (WHO-FC II–III), who have not received PAH medication treatment or are currently receiving stable doses of non-endothelin receptor antagonist (non-ERA) PAH medications for at least 4 weeks, were enrolled in this study. All subjects must have a baseline 6-min walk distance of at least 150 m. Right heart catheterization (RHC) has been performed within 6 months prior to screening and meets the following hemodynamic criteria: mean pulmonary artery pressure (mPAP) ≥25 mmHg, pulmonary vascular resistance (PVR) ≥3 wood units, and pulmonary artery wedge pressure (PAWP) or left ventricular end-diastolic pressure ≤15 mmHg. Pulmonary function tests have been performed within 6 months prior to screening and meet the following criteria: total lung capacity ≥60% and forced expiratory volume in the first second (FEV1) ≥55% of predicted normal values.

Subjects with serum alanine aminotransferase (ALT) or aspartate aminotransferase (AST) levels >2 × ULN, serum bilirubin levels >1.5 × ULN, severe hepatic insufficiency (Child–Pugh grade C), severe renal insufficiency (creatinine clearance <30 ml/min), hemoglobin concentration <100 g/L or hematocrit <30%, severe hypotension (diastolic blood pressure <50 mmHg or systolic blood pressure <90 mmHg) were excluded. Pregnant and lactating women were excluded.

### Assessment

The primary endpoint for efficacy was Δ6MWD at week 12. The secondary endpoints included Δ6MWD at week 24, changes in the Borg scale, plasma BNP level, WHO-FC, and echocardiogram from baseline to weeks 12 and 24. The echocardiogram measurements include TAPSE (tricuspid annular plane systolic excursion) and TAPSE/PASP (pulmonary arterial systolic pressure). Clinical worsening is defined as the occurrence of all-cause death, hospitalization for PAH deterioration, and change or addition of PAH medications within 24 weeks of treatment duration. The exploratory endpoints were risk stratification improvement, TTCI, and TTCI-low within 24 weeks of treatment duration. TTCI was defined as the time from initiation of treatment to the first occurrence of improvement in at least one level of four-strata risk stratification without clinical worsening. TTCI-low was defined as the time from initiation of treatment to the first occurrence of patients achieving low risk of four-strata risk stratification without clinical worsening (20). All efficacy endpoints were assessed at baseline and every 6 weeks. The safety endpoints were adverse events and their severity, laboratory tests, and liver function.

The four-strata risk stratification was determined in accordance with the latest guideline protocol (1, 21). In brief, 6MWD >440, 320–440, 165–319, and <165 m were respectively scored as 1, 2, 3, and 4 points; WHO-FC I, II, III, and IV were respectively scored as 1, 1, 3, and 4 points; and BNP <50, 50–199, 200–800, and >800 ng/L were, respectively, scored as 1, 2, 3, and 4 points. At least two of the above three variables must be utilized in the risk stratification process, and the rounded average value was the risk point. The final calculated 1, 2, 3, and 4 risk points indicated low, intermediate-low, intermediate-high, and high risk, respectively.

### Data analysis

Assuming a dropout rate of 10% and a study power of 90%, a sample size of 40 patients was required to detect a two-sided test significant difference of an increase in Δ6MWD of 41.8 m with a standard deviation of 83 m after 12 weeks of treatment (6, 18).

This study used the intent-to-treat (ITT) population to describe baseline/demographic data and follow-up efficacy endpoints, including those who received at least one dose of ambrisentan and had baseline measurements and at least one follow-up measurement. First, risk stratification was calculated by raw data of 6MWD, BNP, WHO_FC. Then, the last observation carry forward (LOCF) method of imputation for missing data was used for 6MWD, Borg scale, WHO-FC, risk stratification, TAPSE, and TAPSE/PASP. Subgroup analyses were conducted based on age, gender, PAH classification, WHO-FC, risk, and treatment groups at baseline. Normal variables were described by means (SDs), non-normal variables were described by medians (quartiles), and ordinal variables were described by composition ratios. For comparing baseline and follow-up efficacy endpoints, normal variables were compared by paired *t*-tests and non-normal variables and ordinal variables were compared by Wilcoxon signed-rank tests. TTCI, TTCI-low, and TTCW were analyzed by Kaplan–Meier analysis and univariate/multivariate COX model regression analysis. Two treatment group analyses were performed using two independent-sample *t*-tests to compare normal variables and Mann–Whitney *U* tests to compare non-normal and ordinal variables. All statistical analyses were conducted using SPSS version 26.0. Sample size calculations were performed using PASS version 15.0.

Safety was assessed by the safety population, including only those who received at least one dose of ambrisentan treatment.

## Results

### Patient disposition and demographic/baseline characteristics

**Figure 1:**
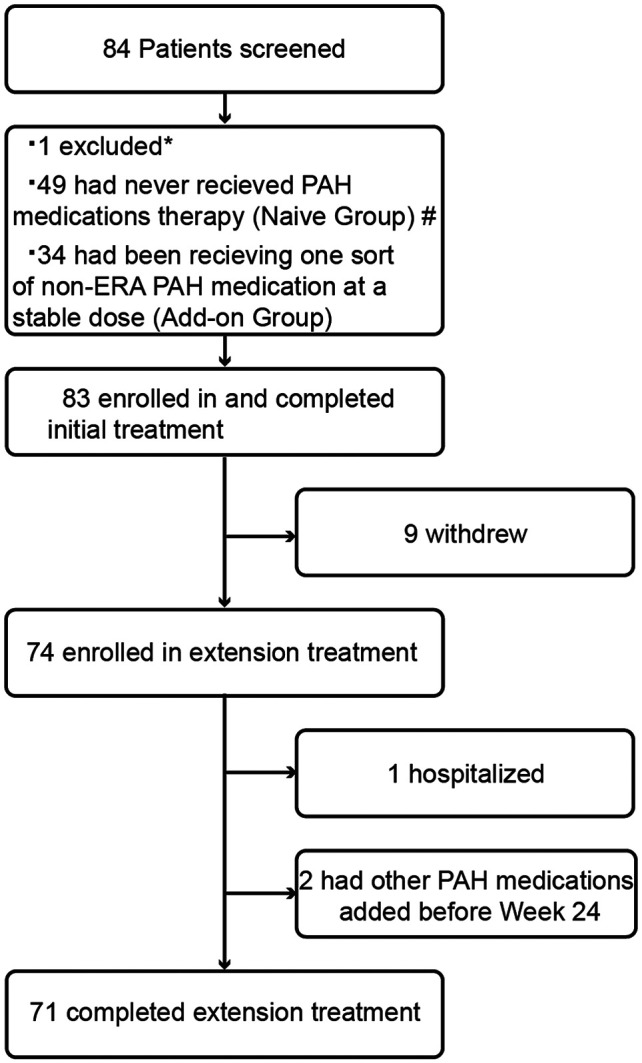
Patient disposition. *: One subject was excluded due to pulmonary hypertension-associated hypoxia; #: one subject was not included in the ITT population due to the lack of baseline efficacy measurements; PAH, pulmonary arterial hypertension; non-ERA PAH medications, including phosphodiesterase 5 inhibitors, guanylate cyclase stimulators, prostacyclin analogues, and prostacyclin receptor agonists.

A total of 83 subjects were successfully enrolled and completed initial treatment. Nine patients withdrew from the study primarily due to drug interruption caused by the COVID-19 pandemic, resulting in 74 subjects entering the extension phase of treatment (Figure 1). The first subject was enrolled in December 2018. The last subject follow-up time was in September 2021. The number of subjects enrolled by each center can be seen in Supplementary Table S1 in the supplementary materials. The average exposure time of all subjects was 160.1 (44.4) days. One subject was excluded from ITT population because of the lack of baseline efficacy endpoint data. Thus, 82 subjects were enrolled in the ITT population, including 48 subjects who had never received PAH medication therapy (naive group) and 34 subjects who had been receiving one sort of non-ERA PAH medication on a stable dose (add-on group).

The demographic/baseline characteristics are shown in Table 1. The study population was primarily women (81.7%), with a median age of 36 years. The baseline 6MWD of the patients was 403.5 (78.0) m. The baseline risk stratification was mostly intermediate risk, with 36 (43.9%) patients at intermediate-low risk, 45 (54.9%) patients at intermediate-high risk, and no subjects at low risk. Sildenafil was the most combined PAH medication in the add-on group (85.3%). Except for age (*P *= 0.034), there were no significant differences in other demographic/baseline characteristics between the naive group and add-on group subjects.

**Table 1:** Demographic and baseline characteristics (ITT population).

	Total (*N *= 82)	Naive group (*n1* = 48)	Add-on group (*n2* = 34)	*P value
Age (years)	36.0 (30.0–43.0)	37.0 (32.0–47.0)	35.0 (28.0–39.3)	0.034
Gender, male/female, *n* (%)	15 (18.3)/67 (81.7)	8 (16.6)/40 (83.3)	7 (20.6)/27 (79.4)	0.802
**Ethnicity, *n* (%)**				
Chinese	82 (100)	48 (100)	34 (100)	
**BMI (kg/m^2^)**	**21.0 (19.1–24.2)**	**21.3 (19.5–25.1)**	**20.3 (19.0–22.3)**	**0.090**
**PAH classification, *n* (%)**				**0.329**
CHD-PAH	25 (30.5)	18 (37.5)	7 (20.6)	
CTD-PAH	36 (43.9)	18 (37.5)	18 (52.9)	
IPAH	21 (25.6)	12 (25.0)	9 (26.5)	
**Right heart catheterization**				
sRVP (mmHg)	72.9 (24.4)	71.7 (26.1)	74.8 (22.0)	0.571
mPAP (mmHg)	48.7 (14.8)	48.0 (16.4)	49.8 (12.3)	0.598
PAWP (mmHg)	8.0 (5.0–12.0)	8.0 (5.0–11.5)	10.0 (7.8–12.0)	0.078
CO (L/min)	4.4 (1.3)	4.5 (1.3)	4.2 (1.3)	0.300
PVR (Wood unit)	9.2 (6.0–12.5)	8.9 (5.6–12.0)	10.0 (6.9–13.8)	0.158
SvO_2_ (%)	67.3 (8.7)	66.8 (8.8)	68.0 (8.5)	0.541
**6MWD (m)**	**403.5 (78.0)**	**416.1 (75.2)**	**385.7 (79.5)**	**0.081**
**Borg scale**	**3.5 (3.0–5.0)**	**3.5 (3.0–4.0)**	**4.0 (2.8–5.0)**	**0.894**
**WHO-FC, *n* (%)**				**0.110**
Class II	40 (48.8)	27 (56.3)	13 (38.2)	
Class III	42 (51.2)	21 (43.8)	21 (61.8)	
**BNP^a^ (ng/L)**	**366.0 (130.5–870.2)**	**513.0 (128.8–972.0)**	**366.0 (128.6–710.0)**	**0.663**
**RISK, *n* (%)**				**0.236**
Low	0	0	0	
Intermediate-low	36 (43.9)	24 (50.0)	12 (35.3)	
Intermediate-high	45 (54.9)	23 (47.9)	22 (64.7)	
High	1 (1.2)	1 (2.1)	0	
**Echocardiogram** ^b^				
TAPSE (mm)	16.0 (13.0–18.0)	16.0 (14.0–18.0)	16.0 (12.0–17.0)	0.290
PASP (mmHg)	80.0 (24.6)	80.6 (24.4)	79.1 (25.3)	0.797
TAPSE/PASP (mm/mmHg)	0.200 (0.154–0.275)	0.194 (0.161–0.254)	0.206 (0.139–0.281)	0.996
**Drug combination, *n* (%)**				
Ambrisentan monotherapy	48 (58.5)	48 (100)	0	
With sildenafil	29 (35.4)	0	29 (85.3)	
With tadalafil	3 (3.7)	0	3 (8.8)	
With beraprost	2 (2.4)	0	2 (5.9)	

ITT population, intent-to-treat population, including those who received at least one dose of ambrisentan and had baseline measurements and at least one follow-up measurement; BMI, body mass index; CHD-PAH, pulmonary arterial hypertension associated with congenital heart disease; CTD-PAH, pulmonary arterial hypertension associated with connective tissue disease; IPAH, idiopathic pulmonary arterial hypertension; sRVP, systolic right ventricular pressure; mPAP, mean pulmonary arterial pressure; PAWP, pulmonary arterial wedge pressure; CO, cardiac output; PVR, pulmonary vascular resistance; SvO_2_, mixed venous oxygen saturation; 6MWD, 6-min walk distance; WHO-FC, World Health Organization cardiac functional class; BNP, plasma B-type natriuretic peptide; TAPSE, tricuspid annular plane systolic excursion; PASP, pulmonary arterial systolic pressure.

* *P *< 0.05 indicates a statistically significant difference between the naive group and the add-on group.

^a^ *N *= 77, *n1 *= 48, *n2 *= 29.

^b^ *N* = 78, *n1 *= 48, *n2 *= 30.

### Δ6MWD and the other traditional efficacy endpoints

**Figure 2:**
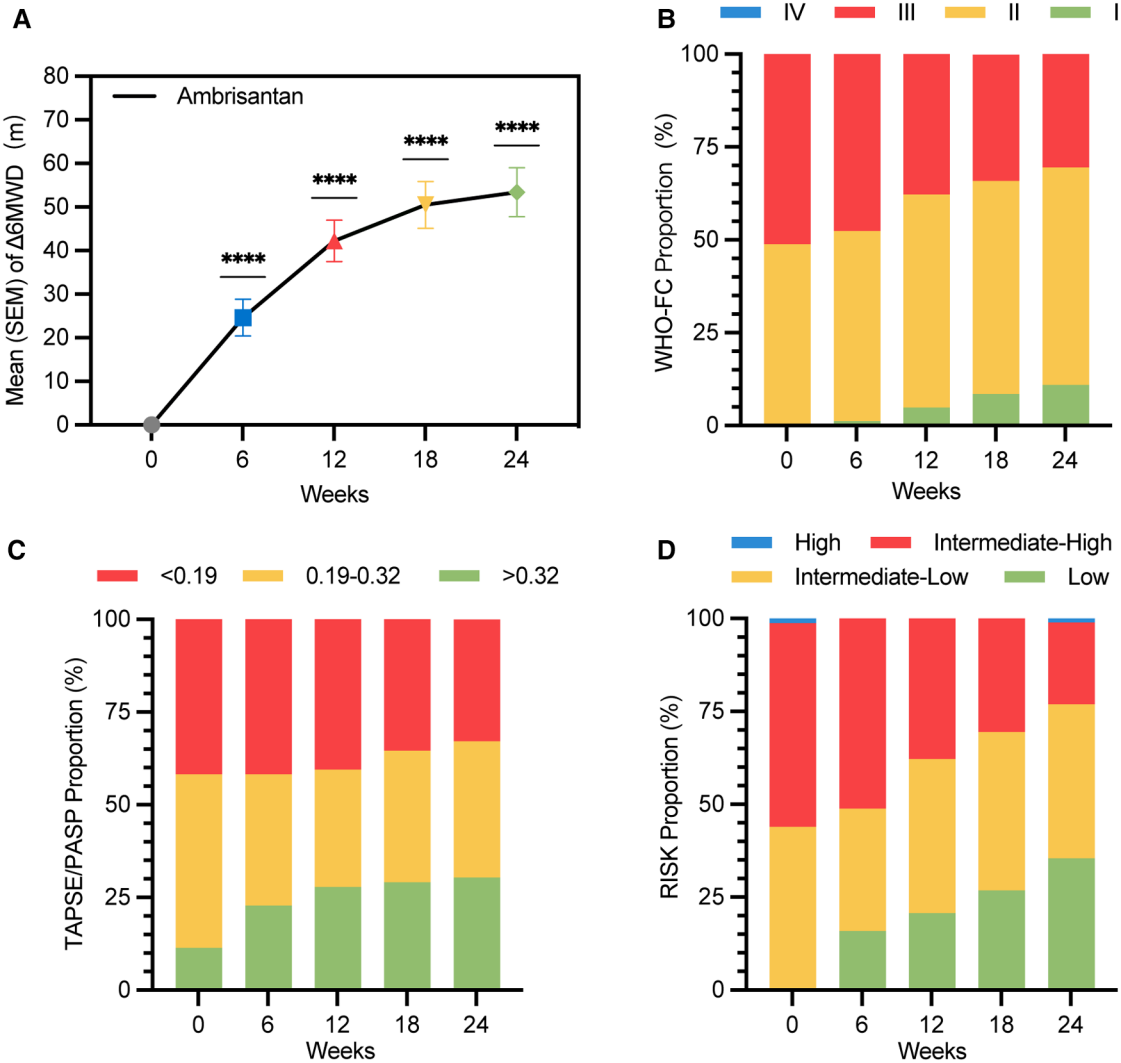
Improvement in Δ6MWD, WHO-FC, TASPE/PASP, and risk stratification over 24 weeks of treatment duration (LOCF) (ITT population). (**A**) Mean (SEM) values of Δ6MWD are 24.6 (4.2) m, 42.2 (4.7) m, 50.5 (5.3) m, and 53.4 (5.6) m at weeks 6, 12, 18, and 24, respectively (****P < 0.0001); (**B**) WHO-FC proportions at week 24 were I (11.0%), II (58.5%), and III (30.5%). A total of 25 (30.5%) subjects improved in at least one level of WHO-FC at week 24; (**C**) TAPSE/PASP proportions at week 24 were <0.19 (32.9%), 0.19–0.32 (36.7%), and >0.32 (30.4%). A total of 26 (32.9%) subjects improved in at least one level of TAPSE/PASP at week 24; (**D**) risk stratification proportions at week 24 were low (35.4%), intermediate-low (41.5%), intermediate-high (22.0%), and high (1.2%). A total of 53 (64.6%) subjects improved in at least one level of four-strata risk stratification within 24 weeks without clinical worsening; Δ6MWD: change in 6MWD from baseline to follow-up time; WHO-FC, World Health Organization cardiac functional class.

A significant improvement in Δ6MWD was observed since week 6, with further significant clinical improvement at week 12 (42.2 m, *P *< 0.0001) and week 24 (53.4 m, *P *< 0.0001). As illustrated in Figure 2 and Table 2, changes in the Borg scale, WHO-FC, BNP, TAPSE, and TAPSE/PASP all demonstrated significant improvements at weeks 12 and 24, which was consistent with Δ6MWD. The result of subgroup analyses of Δ6MWD was similar to the pattern noted in the overall population. Also, there were no statistical differences in Δ6MWD between subgroups (Supplementary Table S2 in the supplementary material). There were no statistical differences in Δ6MWD at week 24 between the naive group (50.9 m) and the add-on group (56.9 m, *P* = 0.609). Additionally, there were no significant differences in other traditional efficacy endpoints between the two groups (Table 2).

**Table 2:** Change in efficacy endpoints from baseline after ambrisentan treatment (ITT population).

Efficacy endpoints	Week 12 (*N1 *= 82)	Week 24 (*N2 *= 82)	Week 24		
			Naive group (*n1 *= 48)	Add-on group (*n2 *= 34)	*P value
**6MWD (m) (LOCF)**					
Mean (SD)	445.7 (78.0)	456.9 (76.5)	467.1 (75.0)	442.6 (77.4)	
Δ6MWD, mean (SD)	42.2 (42.9)	53.4 (50.9)	50.9 (50.2)	56.9 (52.5)	0.609
**P value	<0.0001	<0.0001	<0.0001	<0.0001	
**Borg scale (LOCF)**					
Median (quartiles)	3.5 (2.8–4.0)	3.0 (2.0–4.0)	3.0 (3.0–4.0)	2.5 (2.0–3.6)	
Descended from baseline, median (quartiles)	0.0 (0.0–1.0)	0.5 (0.0–1.0)	0.0 (0.0–1.0)	1.0 (0.0–2.0)	0.115
**P value	<0.001	<0.0001	<0.0005	<0.0005	
**WHO-FC, *n* (%) (LOCF)**					
Improved by class 1/2	17 (20.7)/0	22 (26.8)/3 (3.7)	14 (29.2)/0	8 (23.5)/3 (8.8)	
No change	63 (76.8)	55 (67.1)	33 (68.8)	22 (64.7)	0.635
Worsened by class 1/2	2 (2.4)/0	2 (2.4)/0	1 (2.1)/0	1 (2.9)/0	
**P value	0.001	<0.0001	0.001	0.005	
**BNP^a^ (ng/L) (observed data)**					
Median (quartiles)	167.0 (45.5–487.0)	95.0 (37.8–268.3)	109.0 (37.0–356.0)	93.0 (37.5–254.5)	
Descended from baseline, median (quartiles)	136.0 (22.0–632.5)	130.0 (12.5–610.3)	175.0 (14.0–440.0)	115.0 (2.0–671.0)	0.989
**P value	<0.0001	<0.0001	0.003	0.003	
**TAPSE^b^ (mm) (LOCF)**					
Median (quartiles)	16.0 (14.0–18.0)	16.0 (14.0–18.0)	0.232 (0.172–0.377)	0.266 (0.185–0.339)	
Improved from baseline, median (quartiles)	1.0 (0.0–2.0)	1.0 (−1.0 to 2.0)	0.040 (0.000–0.091)	0.045 (0.002–0.090)	0.698
**P value	0.008	0.003	0.095	0.003	
**TAPSE/PASP^b^ (mm/mmHg) (LOCF)**					
Median (quartiles)	0.231 (0.173–0.333)	0.235 (0.174–0.370)	0.232 (0.172–0.377)	0.266 (0.185–0.339)	
Improved from baseline, median (quartiles)	0.019 (0.000–0.079)	0.042 (0.000–0.088)	0.040 (0.000–0.091)	0.045 (0.002–0.090)	0.585
**P value	<0.0001	<0.0001	<0.0001	<0.0005	
Achieved low risk, *n* (%) (LOCF)	17 (20.7)	29 (35.4)	21 (43.8)	8 (23.5)	0.061
**RISK improvement, *n* (%) (LOCF)**					
Improved by class 1/2	27 (32.9)/3 (3.7)	48 (58.5)/5 (6.1)	35 (72.9)/2 (4.2)	13 (38.2)/3 (8.8)	
No change	52 (63.4)	27 (32.9)	9 (18.8)	18 (52.9)	0.043
Worsened by class 1/2	0/0	2 (2.4)/0	2 (4.2)/0	0/0	
**P value	<0.0001	<0.0001	<0.0001	<0.0005	
**Clinical improvement^c^**					
*n* of events (%)		53 (64.6)	37 (77.1)	16 (47.1)	
Median improvement time (95% CI) (days)	131.0 (125.3–136.7)	94.0 (55.6–132.4)	173.0 (121.4–224.6)		
Cumulative improvement rate (SEM) (%)	75.1 (0.061)	84.1 (0.060)	57.9 (0.119)		
*Log-rank *P* value			0.008		

Δ6MWD, change in 6MWD from baseline to follow-up time; other abbreviations as in Table 1.

* *P* < 0.05 indicates a statistically significant difference between the naive group and the add-on group.

** *P* < 0.05 indicates a statistically significant difference between baseline and the follow-up measurement.

^a^ *N1 *= 61, *N2 *= 40, *n1 *= 19, *n2 *= 21.

^b^ *N1 *= 79, *N2 *= 79, *n1 *= 49, *n2 *= 30.

^c^ Clinical improvement, defined by reaching an improvement in at least one level of four-strata risk stratification without clinical worsening.

### Risk improvement and time to clinical improvement

**Figure 3:**
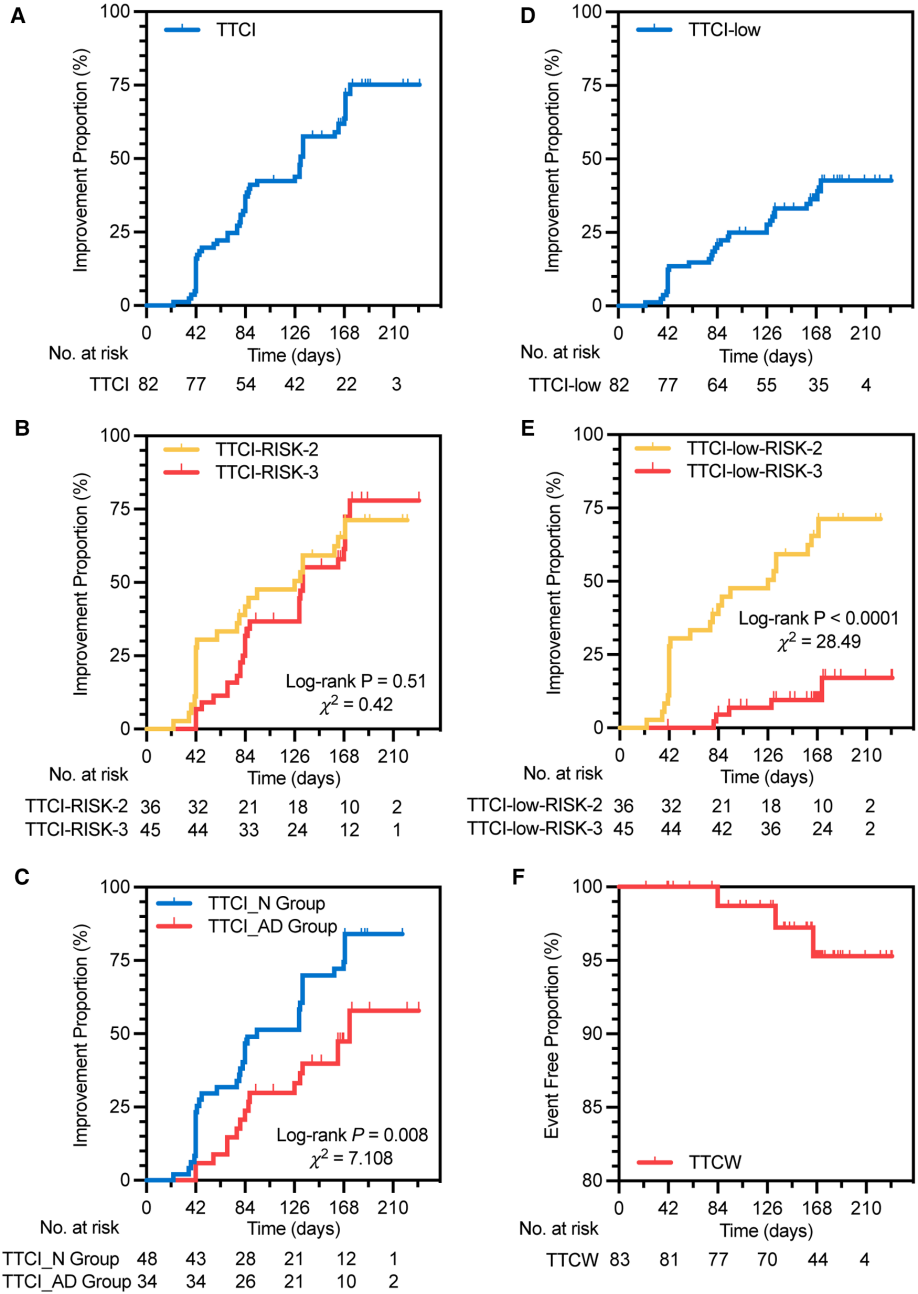
Kaplan–Meier analysis of TTCI (**A**), TTCI in two risk groups (**B**), TTCI in two treatment groups (**C**), TTCI-low (**D**), TTCI-low in two risk groups (**E**), and TTCW (**F**). TTCI, time to clinical improvement, is defined as the time from initiation of treatment to the first occurrence of improvement in at least one level of four-strata risk stratification within 24 weeks of treatment duration without clinical worsening; RISK-2, intermediate-low risk; RISK-3, intermediate-high risk; TTCI_N group, TTCI of the naive group, in which subjects had never received PAH medication therapy; TTCI_AD group, TTCI of the add-on group, in which subjects had been receiving one sort of non-ERA PAH medication on a stable dose; TTCI-low, defined as the time from initiation of treatment to the first occurrence of patients achieving low risk of four-strata risk stratification within 24 weeks of treatment duration without clinical worsening; TTCW, time to clinical worsening, defined as the time from initiation of treatment to the first occurrence of all-cause death from baseline, hospitalization for PAH deterioration, and change or addition of PAH medications within 24 weeks of treatment duration.

A total of 29 (35.4%) subjects achieved low-risk status within 24 weeks without clinical worsening (Figure 2D). Kaplan–Meier analysis of TTCI-low revealed that the cumulative improvement rate was 41.4% within 24 weeks (Figure 3D). Subjects in the intermediate-low-risk group reached low-risk status significantly earlier than those in the intermediate-high-risk group (log-rank *P* < 0.0001, Figure 3E).

Univariate COX model regression analysis showed TTCI-low was associated with Δ6MWD at week 24 and most of the baseline characteristics, including RHC (sRVP, mPAP, PVR, SvO_2_), 6MWD, WHO-FC, BNP, echocardiogram (TAPSE, PASP, TAPSE/PASP), and risk stratification at baseline (Supplementary Table S3 in the supplementary material).

A total of 30 (36.6%) and 53 (64.6%) subjects improved in at least one level of four-strata risk stratification within 12 and 24 weeks without clinical worsening (Table 2). Kaplan–Meier analysis of TTCI revealed that the median improvement time was 131 (95% CI: 125.4–136.7) days, and the cumulative improvement rate was 75.1% (Figure 3A and Table 2). There was no significant difference in TTCI between intermediate-low-risk subjects and intermediate-high-risk subjects (median improvement time was 133 (95% CI: 73.9–178.1) days vs. 126 (95% CI: 128.6–137.4) days, log-rank *P* = 0.51, Figure 3B). TTCI of naive group patients is shorter than that of the add-on group patients [median improvement time and cumulative improvement rate were respectively 94.0 (95% CI: 55.6–132.4) days and 84.1% vs. 173.0 (95% CI: 121.4–224.6) days and 57.9%, log-rank *P* = 0.008, Figure 3C and Table 2].

Univariate COX model regression analysis showed that TTCI was associated with the treatment group, Δ6MWD at week 24, change in WHO-FC at week 24, and PASP and TAPSE/PASP at baseline. After adjustment for these above variables, the treatment group, Δ6MWD, and change in WHO-FC at week 24 were independent influencing factors of TTCI, with hazard ratios of 0.314 (95% CI: 0.164–0.603, *P* < 0.001), 1.009 (95% CI: 1.002–1.016, *P* = 0.017), and 2.974 (95% CI: 1.703–5.191, *P* < 0.001), respectively (Table 3).

**Table 3:** Results of univariate and multivariate Cox model regression analyses of TTCI.

	Univariate regression			Multivariate regression		
	Hazards ratio	95% CI	*P* value	Hazards ratio	95% CI	*P* value
**Demographic/baseline characteristics**						
Age	1.011	0.987–1.035	0.393			
Gender	0.706	0.332–1.499	0.365			
Treatment group^a^	0.467	0.259–0.840	0.011	0.314	0.164–0.603	<0.001
PAH classification	0.953	0.665–1.366	0.794			
BMI	0.978	0.905–1.056	0.569			
sRVP	0.993	0.982–1.005	0.254			
mPAP	0.986	0.967–1.005	0.146			
PAWP	1.012	0.942–1.088	0.737			
CO	1.085	0.895–1.315	0.406			
PVR	0.954	0.906–1.006	0.080			
SvO2	1.020	0.989–1.051	0.211			
6MWD	1.002	0.998–1.005	0.316			
Borg scale	1.028	0.851–1.241	0.773			
WHO-FC	0.733	0.427–1.257	0.259			
BNP	1.000	0.9996–1.0003	0.711			
TAPSE	1.050	0.968–1.138	0.238			
PASP	0.986	0.974–0.998	0.024	0.990	0.972–1.008	0.291
TAPSE/PASP	69.121	2.560–1,866.541	0.012	0.415	0.003–62.777	0.731
RISK	0.917	0.536–1.568	0.751			
**Change from baseline to week 24:**						
6MWD	1.010	1.004–1.016	0.001	1.009	1.002–1.016	0.017
Borg scales	1.017	0.787–1.315	0.895			
WHO-FC	2.675	1.749–4.090	<0.0001	2.974	1.703–5.191	<0.001
BNP	1.000	0.999–1.001	0.551			
TAPSE	1.026	0.931–1.130	0.602			
PASP	0.998	0.986–1.011	0.807			
TAPSE/PASP	4.414	0.295–65.920	0.282			

All abbreviations as in Tables 1, 2.

^a^ Naive group or add-on group.

### Time to clinical worsening and adverse events

Three (3.6%) subjects experienced clinical worsening events. One was hospitalized for PAH, two added other PAH medications within the study duration (one added sildenafil and one added tadalafil), and no patient experienced all-cause death or lung transplantation within the study duration (Table 4 and Figure 3F).

**Table 4:** Clinical worsening and most frequent (≥3%) adverse events during ambrisentan treatment (safety population) by maximum intensity.

Events	Ambrisentan (*N *= 83)
**Clinical worsening events, *n* (%)**	3 (3.6)
Death	0
Hospitalization for worsening PAH	1 (1.2)
Add or change PAH medications	2 (2.4)
**Adverse events, *n* (%)**	45 (54.2)
Flushing	12 (14.5)
Palpitation	8 (9.6)
Oedema peripheral	7 (8.4)
Headache	6 (7.2)
Nasopharyngitis	6 (7.2)
Aspartate aminotransferase increased	4 (4.8)
Alanine aminotransferase increased^a^	3 (3.6)
Diarrhea	3 (3.6)
Constipation	3 (3.6)

^a^ One subject had >3 × ULN elevation in alanine aminotransferase enzymes (ALT); all the other adverse events were mild to moderate.

A total of 45 (54.2%) subjects experienced adverse events. The most common adverse events were flushing (14.5%), palpitation (9.6%), and peripheral edema (8.4%). No significant changes were observed in hematological parameters. One patient withdrew from the study due to a > 3 × ULN elevation in alanine aminotransferase (ALT) enzymes (Table 4), and all the other adverse events were mild to moderate in severity.

## Discussion

In this study, we demonstrated that domestic ambrisentan significantly increased Δ6MWD and ameliorated the risk status of Chinese PAH patients. Also, we observed the characteristics of TTCI and TTCI-low, which shows that TTCI is more suitable than TTCI-low for use as a surrogate endpoint for PAH trials.

Since Barst first used Δ6MWD as the primary endpoint to assess the efficacy of epoprostenol in 1990s (22), most PAH medication studies have followed this criterion until 2010 (17, 18, 23–25). There was no clearly defined cutoff value for Δ6MWD effectiveness until 2012 when a meta-analysis synthesized the last 10 RCTs of PAH medication and concluded that an improvement of 41.8 m could be considered a cutoff value for less clinical worsening events within 12 weeks (6). The increase of 42.2 (42.9) m at week 12 and 53.4 (50.9) m at week 24 in our study indicated that domestic ambrisentan is effective in treating Chinese adults with PAH (Table 2 and Figures 2A–C). Moreover, the result of subgroup analyses based on age, gender, PAH classification, WHO-FC, and risk at baseline was similar to the pattern noted in the overall population. Additionally, there were no statistical differences in Δ6MWD between subgroups (Supplementary Table S2 in the supplementary material). Other secondary endpoints were consistent with Δ6MWD. Most of the adverse events were moderate to mild, consistent with the results of peer studies (18), indicating that domestic ambrisentan was well tolerated in treating Chinese adults with PAH (Table 4).

Risk improvement is a composite endpoint. Hoeper et al. validated that subjects who improved in risk status had a better survival rate than those who did not improve (16). Also, their study showed that 35.2% and 51% of subjects improved in WHO-FC and risk status, respectively, within 3–12 months (16). In our study, 25 (30.5%) and 53 (64.6%) subjects improved in WHO-FC and at least one level of four-strata risk stratification within 24 weeks, which is consistent with the trend of the Hoeper’s study. Domestic ambrisentan significantly improved the risk status of Chinese adults with PAH (*P* < 0.0001, Table 2 and Figure 2D).

Our study may be the first prospective study to combine clinical improvement time and risk stratification tools as surrogate endpoints. In this study, we used two ways to implement this idea. The first one is to define the clinical improvement event by reaching low-risk status of four-strata risk stratification (TTCI-low). The second one is to define clinical improvement by reaching an improvement in at least one level of four-strata risk stratification (TTCI). Similar to TTCW, maybe the limited occurrence of positive events will also be a restriction for TTCI and TTCI-low in short-term trials. Also, a relatively high rate of positive events is advantageous for reducing the sample size requirement (16, 26). Our study shows that, within 24 weeks, the positive events of TTCI and TTCI-low were 53 (64.6%) and 29 (35.4%), respectively, with cumulative improvement rates of 75.1% and 41.4%, respectively (Figures 3A,D). This result indicated that, compared to TTCI-low, TTCI has the advantages of requiring a smaller sample size and a shorter follow-up time.

Univariate Cox model regression analysis showed that TTCI-low can vary greatly due to the baseline status of the study population. This factor will increase the difficulty of matching patients' baseline conditions between groups when assessing the efficacy of different PAH treatment regimens (Figure 3E and Supplementary Table S3 in the supplementary material). On the contrary, TTCI was not associated with risk stratification, 6MWD, WHO-FC, or BNP levels in subjects’ baseline status, indicating that the occurrence of improvement event was largely due to the treatment itself (Table 3). Previous studies have shown that 6MWD has a “ceiling effect,” which means that in individuals with a higher baseline value of 6MWD, lower Δ6MWD tends to be observed in the overall population after treatment (27–29). TTCI is not affected by baseline risk status (Figure 3B), which is superior to Δ6MWD and TTCI-low.

Multivariate Cox model regression analysis showed that the treatment groups, Δ6MWD, and change in WHO-FC at week 24 were independent influencing factors of TTCI (Table 3). Our study showed that naive group patients have shorter TTCI (median improvement time was 94 vs. 173 days, log-rank *P* = 0.008, Figure 3C) and more risk improvement (*P* = 0.043, Table 2) than add-on group patients, while Δ6MWD and the other traditional endpoints at week 24 did not show significant differences between two groups. This result implied that TTCI could identify the difference in treatment efficacy which is not detectable using Δ6MWD or the other traditional efficacy endpoints alone. However, it needs further study to prove.

It must be pointed out that the naive group exhibiting shorter TTCI does not conflict with the clinical recommendation for combination therapy. A similar result was observed by McLaughlin et al. (30). Compared to monotherapy with sildenafil, they found that adding bosentan to sildenafil treatment did not result in a better amelioration in clinical worsening time. This is possible because subjects in the add-on group had previously received stable doses of PAH medications and some of them had already experienced clinical improvement, leading to fewer occurrences of improvement events when the ambrisentan was added.

## Limitations

Our study has several limitations. First, it was an open-label, single-arm study without a placebo control group due to the availability of other medications for treating PAH. Second, the sample size was relatively small, which limited the number of baseline characteristics that could be included in the multivariate analysis and discussion. Third, the follow-up duration was relatively short, which hindered a thorough investigation of the relationship between risk improvement and the long-term survival of patients. To further clarify these issues, larger randomized controlled trials using TTCI as an exploratory surrogate endpoint are needed. In future studies, it may also be possible to determine specific cutoff values for the effective median improvement time for PAH medication therapy based on the relationship between TTCI and the mortality of PAH patients.

## Conclusion

Domestic ambrisentan significantly improved the exercise capacity and risk status of Chinese adults with PAH. Compared to TTCI-low, TTCI has a relatively high positive rate within 24 weeks and is not affected by most of the baseline status. Also, TTCI could identify the difference in treatment efficacy, which is not detected by Δ6MWD. Thus, TTCI is an appropriate composite surrogate endpoint for PAH medication.

## Data Availability

The original contributions presented in the study are included in the article/**Supplementary Material**; further inquiries can be directed to the corresponding author.
