# Corrigendum: Time to clinical improvement: an appropriate surrogate endpoint for pulmonary arterial hypertension medication trials

**DOI:** 10.3389/fcvm.2024.1371699

**Published:** 2024-02-21

**Authors:** An Wang, Mengqi Chen, Qi Zhuang, Lihua Guan, Weiping Xie, Lan Wang, Wei Huang, Zhaozhong Cheng, Shiyong Yu, Hongmei Zhou, Jieyan Shen

**Affiliations:** ^1^ Department of Cardiology, Renji Hospital, School of Medicine, Shanghai Jiao Tong University, Shanghai, China; ^2^ Department of Cardiology, Shanghai Institute of Cardiovascular Disease, Zhongshan Hospital, Fudan University, Shanghai, China; ^3^ Department of Respiratory and Critical Care Medicine, The First Affiliated Hospital of Nanjing Medical University, Nanjing, China; ^4^ Department of Cardio-Pulmonary Circulation, Shanghai Pulmonary Hospital, School of Medicine, Tongji University, Shanghai, China; ^5^ Department of Cardiology, The First Affiliated Hospital of Chongqing Medical University, Chongqing, China; ^6^ Respiratory Department, The Affiliated Hospital of Qingdao University, Qingdao, China; ^7^ Department of Cardiology, The Second Affiliated Hospital, Third Military Medical University (Army Medical University), Chongqing, China; ^8^ Congenital Heart Disease Center, Wuhan Asia Heart Hospital, Wuhan University of Science and Technology, Wuhan, China

**Keywords:** pulmonary arterial hypertension, time to clinical improvement, efficacy, risk stratification, 6-min walk distance

A Corrigendum on Time to clinical improvement: an appropriate surrogate endpoint for pulmonary arterial hypertension medication trials By Wang A, Chen M, Zhuang Q, Guan L, Xie W, Wang L, Huang W, Cheng Z, Yu S, Zhou H, Shen J. (2023). Front Cardiovasc Med. 10:1142721. doi: 10.3389/fcvm.2023.1142721

**Incorrect Funding**

In the published article, there was an error in the Funding statement.

There was an error in specifying the grant number of National Natural Science Foundation of China (CN) in the Funding section of the paper. The correct grant number is [81970047] and [82270050], but it was mistakenly written as [81800047].

The original text was: This work was supported by the National Natural Science Foundation of China (CN) (81800047) and the Clinical Research Innovation and Incubation Foundation of Renji Hospital, Shanghai Jiao Tong University School of Medicine (PYII20-13).

The correct Funding statement appears below.

