# Supplementary material for: Time to clinical improvement: an appropriate surrogate endpoint for pulmonary arterial hypertension medication trials
Source: Front Cardiovasc Med. 2023 Jun 12;10:1142721. doi: 10.3389/fcvm.2023.1142721 (PMC10291317; doi:10.3389/fcvm.2023.1142721)
Supplement: Supplementary file 1 [file Datasheet1.pdf]

Supplementary Material

Time to Clinical Improvement: an appropriate surrogate endpoint for pulmonary arterial hypertension medication trials

An Wang[1†], Mengqi Chen[1†], Qi Zhuang[1], Lihua Guan[2], Weiping Xie[3], Lan Wang[4], Wei Huang[5], Zhaozhong Cheng[6], Shiyong Yu[7], Hongmei Zhou[8], Jieyan Shen[1*]

[1] Department of Cardiology, Renji Hospital, School of Medicine, Shanghai Jiao Tong University, Shanghai, China.

[2] Department of Cardiology, Shanghai Institute of Cardiovascular Disease, Zhongshan Hospital, Fudan University, Shanghai, China.

[3] Department of Respiratory and Critical Care Medicine, The First Affiliated Hospital of Nanjing Medical University, Nanjing, Jiangsu 210029, P.R. China.

[4] Department of Cardio-Pulmonary Circulation, Shanghai Pulmonary Hospital, School of Medicine, Tongji University, Shanghai, China.

[5] Department of Cardiology, The First Affiliated Hospital of Chongqing Medical University, Chongqing, China.

[6] Respiratory Department, The Affiliated Hospital of Qingdao University, Qingdao, China.

[7] Department of Cardiology, The Second Affiliated Hospital, Third Military Medical University (Army Medical University), Chongqing, China.

[8] Congenital Heart Disease Center, Wuhan Asia Heart Hospital, Wuhan University of Science and Technology, Wuhan, China.

*** Correspondence:**
Jieyan Shen
shenjy_66@163.com

[†]These authors have contributed equally to this work

Table S1. The number of subjects enrolled by each center.

Centers *	*n*, (%)
1. Shanghai Renji Hospital	35 (42.2)
2. Shanghai Zhongshan Hospital	16 (19.3)
3. The First Affiliated Hospital of Nanjing Medical University	9 (10.8)
4. Shanghai Pulmonary Hospital	6 (7.2)
5. The First Affiliated Hospital of Chongqing Medical University	5 (6.0)
6. The Affiliated Hospital of Qingdao University	6 (7.2)
7. The Second Affiliated Hospital, Third Military Medical University	4 (4.8)
8. Wuhan Asia Heart Hospital	2 (2.4)
**Total**	83 (100)

*: The full names of each center can be found in the list of author affiliations.

Table S2. Subgroup analyses of Δ6MWD at week 24 (ITT population)

Subgroups	*n* (%)	**Δ6MWD** (m), mean (SD)	* *P* valuve	# *P* valuve
**Age**, (year)				0.486
18-35	40 (48.8)	58.6 (49.7)	< 0.0001	
36-50	30 (36.6)	52.5 (56.7)	< 0.0001	
51-75	12 (14.6)	38.4 (39.0)	0.0058	
**Gender**				0.124
Male	15 (18.3)	73.9 (55.7)	0.0002	
Female	67 (81.7)	48.8 (49.1)	< 0.0001	
**PAH-Classification**				0.309
CHD-PAH	25 (30.5)	40.9 (40.0)	< 0.0001	
CTD-PAH	36 (43.9)	61.2 (43.4)	< 0.0001	
IPAH	21 (25.6)	55.0 (70.7)	0.0019	
**WHO-FC**				0.324
Class II	40 (48.8)	48.0 (52.4)	< 0.0001	
Class III	42 (51.2)	59.1 (49.3)	< 0.0001	
**RISK**				0.441
Intermediate-low risk	36 (43.9)	50.2 (50.9)	< 0.0001	
Intermediate-high risk	45 (54.9)	59.1 (50.9)	< 0.0001	
**Treatment groups**				0.609
Naive group	48 (58.5)	50.9 (50.2)	< 0.0001	
Add-on group	34 (41.5)	56.9 (52.5)	< 0.0001	

*: Pared t-test, P < 0.05 indicated statistically significant difference between baseline and week 24; #: One-way ANOVA, P < 0.05 indicated statistically significant difference between subgroups. *All abbreviations as in **Tables 1, 2**.*

Table S3. Univariate Cox model regression analysis of TTCI-low

	Univariate Regression		
	**Hazard Ratio**	**95%CI**	***P* value**
**Demographic / Baseline characteristics:**			
Age	0.997	0.965-1.031	0.881
Gender	0.475	0.144-1.570	0.222
Treatment group *	0.448	0.198-1.012	0.053
PAH-Classification	0.852	0.519-1.398	0.526
BMI	0.954	0.859-1.059	0.378
sRVP	0.964	0.945-0.984	0.001
mPAP	0.941	0.911-0.973	< 0.0005
PAWP	0.989	0.898-1.089	0.818
CO	1.186	0.905-1.554	0.217
PVR	0.884	0.808-0.968	0.008
SvO2	1.070	1.024-1.119	0.003
6MWD	1.005	1.000-1.010	0.040
Borg Scale	0.856	0.652-1.125	0.265
WHO-FC	0.243	0.104-0.570	0.001
BNP	0.998	0.997-0.999	0.002
TAPSE	1.142	1.031-1.265	0.011
PASP	0.973	0.955-0.991	0.004
TAPSE/PASP	4247.727	70.149-257212.081	< 0.0001
RISK	0.113	0.043-0.296	< 0.0001
**Change from baseline to week 24:**			
6MWD	1.010	1.003-1.017	0.008
Borg Scales	0.836	0.579-1.207	0.340
WHO-FC	1.679	0.971-2.963	0.063
BNP	0.999	0.997-1.000	0.164
TAPSE	1.032	0.901-1.181	0.652
PASP	0.998	0.979-1.017	0.825
TAPSE/PASP	53.597	1.833-1566.979	0.021

*****: naive group or add-on group; *All abbreviations as in **Tables 1, 2**.*
